# *Drosophila* Photoreceptor Cells Exploited for the Production of Eukaryotic Membrane Proteins: Receptors, Transporters and Channels

**DOI:** 10.1371/journal.pone.0018478

**Published:** 2011-04-08

**Authors:** Valérie Panneels, Ines Kock, Jacomine Krijnse-Locker, Meriem Rezgaoui, Irmgard Sinning

**Affiliations:** 1 Department of Structural Biology, Heidelberg University Biochemistry Center (BZH), Heidelberg, Germany; 2 Department of Infectious Diseases and Core Facility Electron Microscopy (EMCF), University of Heidelberg, Heidelberg, Germany; Universidade Federal do Rio de Janeiro, Brazil

## Abstract

**Background:**

Membrane proteins (MPs) play key roles in signal transduction. However, understanding their function at a molecular level is mostly hampered by the lack of protein in suitable amount and quality. Despite impressive developments in the expression of prokaryotic MPs, eukaryotic MP production has lagged behind and there is a need for new expression strategies. In a pilot study, we produced a *Drosophila* glutamate receptor specifically in the eyes of transgenic flies, exploiting the naturally abundant membrane stacks in the photoreceptor cells (PRCs). Now we address the question whether the PRCs also process different classes of medically relevant target MPs which were so far notoriously difficult to handle with conventional expression strategies.

**Principal Findings:**

We describe the homologous and heterologous expression of 10 different targets from the three major MP classes - G protein-coupled receptors (GPCRs), transporters and channels in *Drosophila* eyes. PRCs offered an extraordinary capacity to produce, fold and accommodate massive amounts of MPs. The expression of some MPs reached similar levels as the endogenous rhodopsin, indicating that the PRC membranes were almost unsaturable. Expression of endogenous rhodopsin was not affected by the target MPs and both could coexist in the membrane stacks. Heterologous expression levels reached about 270 to 500 pmol/mg total MP, resulting in 0.2–0.4 mg purified target MP from 1 g of fly heads. The metabotropic glutamate receptor and human serotonin transporter - both involved in synaptic transmission - showed native pharmacological characteristics and could be purified to homogeneity as a prerequisite for further studies.

**Significance:**

We demonstrate expression in *Drosophila* PRCs as an efficient and inexpensive tool for the large scale production of functional eukaryotic MPs. The fly eye system offers a number of advantages over conventional expression systems and paves the way for in-depth analyses of eukaryotic MPs that have so far not been accessible to biochemical and biophysical studies.

## Introduction

Membrane proteins (MPs) represent more than 30% of the cell proteome [1] and play key roles in signal transduction. Dysfunction often leads to major disorders or death and therefore, MPs account for more than 50% of the current drug targets [2]. However, drug discovery as well as detailed biochemical and structural studies are still hindered by a number of problems already encountered in the production of eukaryotic MPs. It is therefore not surprising that the majority of eukaryotic MPs found in the structural database (Membrane Proteins of Known 3D-Structure, http://blanco.biomol.uci.edu) are naturally abundant [3], [4] and that their structures were determined using material from wild-type organisms. Most of them are localized in specialized cells from i.e. the retina for rhodopsin, the lens for aquaporins, the sarcoplasmic reticulum for calcium ATPases and the electric organ of Torpedo for the nicotinic acetylcholine receptor pore. These cells are adapted to the massive production of MPs, which are often densely packed in their respective membrane environment.

In contrast to eukaryotic MPs, our understanding of prokaryotic MPs has tremendously increased in the past decade due to the optimization of bacterial strains and expression tools for MP production [4], as well as by the use of extremophilic organisms (e.g. Archaea) as a source for MPs of increased stability [5]. Bacteria enriched in membranes are widely used for MP expression as they seem to offer increased membrane surface as well as an optimized insertion machinery [6]. The crystal structures of close prokaryotic homologs provided relevant models for many mammalian MPs. However, some eukaryotic MPs which are of prime interest in neuropharmacology, like the sodium-dependent serotonin transporter (SERT or 5HTT), do not have close bacterial homologs [7]. Importantly, differences in the active sites have been observed e.g. in rhodopsin [8] or potassium channels [9] that distinguish the pro- and eukaryotic proteins. The precise architecture of these binding sites can be difficult to model which leads to controversies in the perception of their reaction mechanisms. For MPs regulated by allosteric mechanisms [10], focusing on the ligand binding site is not sufficient. Among G protein-coupled receptors (GPCRs), metabotropic glutamate receptors (mGluRs) are prototypes for allosteric regulation and have been subjected to random high-troughput ligand screens for drug design as well as structure-based virtual screening [11], [12]. Both, high-throughput pharmacological and structural analyses of MPs require amounts of material which are often not provided in sufficient quality and quantity by conventional expression systems.

Eukaryotic cells in culture, like insect cells and yeast are commonly used for the overexpression of eukaryotic MPs [3]. However, a major drawback is the often limited capacity of these cells for trafficking, folding and membrane insertion of the target MPs and therefore, a significant portion of immature MPs remain trapped in internal membranes [13]. In a pilot study, we engineered a transgenic fly overexpressing a recombinant *Drosophila* metabotropic glutamate receptor (DmGluRA) specifically in the eyes [14]. The idea was to target the receptor to the naturally abundant membrane stacks in the photoreceptor cells (PRCs), the rhabdomeres, housing the GPCR-prototype rhodopsin. *Drosophila melanogaster* was chosen because fly genetics offers the possibility of regulating ectopic expression in intensity, kinetics and localization using specific promoters (drivers). The DmGluRA production in fly eyes gave higher yields than the baculovirus overexpression system in Sf9 cells and the receptor was functional. In addition, the purified protein was clearly superior in homogeneity compared to protein obtained from Sf9 membranes [14] which typically suffers from the presence of immature receptors [3]. The receptor could be purified in mg amounts [14] and biochemical analysis suggested cholesterol as an allosteric regulator that switches the receptor to a high affinity state [15]. Recently, the expression protocol was improved by the use of GFP-fusion constructs [16]. However, the question remained whether overexpression in fly eyes would be also applicable to the heterologous expression for MPs like transporters and channels which are often difficult to express in conventional systems.

In this study, we show the exceptional properties of the PRCs in offering seemingly unsaturable membrane space for target MP insertion. We describe the heterologous expression of functional MPs including mammalian GPCRs, neurotransmitter transporters and the channelrhodopsin ChR2. We establish overexpression in fly eyes as a general, efficient and inexpensive method for large scale production of functional eukaryotic MPs and exemplify our findings with an in depth analysis of mGluR5 and SERT.

## Results

### Photoreceptor cells have a large capacity for recombinant MPs

**Figure 1:**
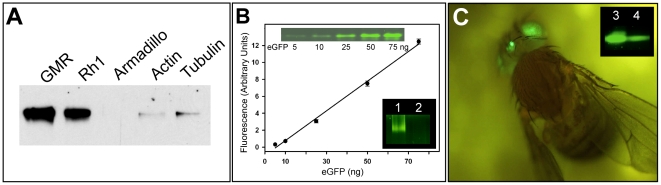
Recombinant expression of *Drosophila* and mammalian GPCRs in fly eyes. (**A**). Western blot analysis of DmGluRA expression using eye-specific (GMR, Rh1) or ubiquitous (Armadillo, Actin, Tubulin) promoter elements. DmGluRA was detected with the 7G11 antibody. β-tubulin was used as a loading control (not shown). (**B**). Quantification of DmGluRA-GFP expressed under the control of the GMR driver. Intrinsic fluorescence signal of DmGluRA-GFP (1) compared with the GMR driver fly (2)(lower inset). The heads of three flies were analyzed. The GFP standard curve shown as graph was obtained by fluorescence scanning of a clear-native gel (using 5, 10, 25, 50, 75 ng GFP; upper inset). Fluorescence signals were integrated with the ImageJ software. (**C**). Typical fluorescence image of a transgenic *Drosophila* expressing the human vasopressin receptor V2R-GFP under the control of the eye-specific GMR^1104^ driver. The inset shows the fluorescence signals of V2R-GFP (3) and Rh1-GFP (4) from three fly heads.

The successful expression of a functional *Drosophila* metabotropic glutamate receptor DmGluRA in fly eyes recommended this system for the production of eukaryotic MPs [14] (see Supporting Information: Primer of the fly eye system (Primer S1)). We now addressed the question whether overexpression in the eyes is superior to overexpression e.g. in the whole fly or other body parts. DmGluRA was expressed in transgenic flies under the control of different drivers [17] inducing specific expression in the eyes (GMR- or Rh1-GAL4) or ubiquitous expression (Tubulin-, Actin- or Armadillo-GAL4). The expression driven by eye-specific promoters was impressive compared to the insignificant levels obtained with ubiquitous promoters (Figure 1A). Using an eye-specific driver was a prerequisite for high expression.

The green fluorescent protein (GFP) was fused to the C-terminus of all MP-targets in this study for efficient monitoring [18], e.g. to select the best expressing flies, for quantification, localization of expression and for quality control of large-scale cultures. GFP fluorescence indicates also correct folding of the N-terminally fused partner protein [19]. Flies expressing different GPCR-GFP fusion constructs under the control of GMR-GAL4 were generated (for technical details see [16]). Quantification by fluorescence-scanning of native gels (Figure 1B) showed that e.g. DmGluRA expression levels reached about 50% of endogenous rhodopsin (Rh1) present in the PRCs (Table 1). Recombinant Rh1 could be expressed at similar levels (502 pmol/mg MP or 18 ng/eye) as endogenous Rh1 (3 to 6×10^7^ Rh1 molecules/rhabdomere, corresponding to 10 to 20 ng/eye [20], [21]) and similar to recombinant Rh1 not fused to GFP (15 ng/eye [22]) (Table 1). A number of rhodopsin-type GPCRs (Class A GPCRs [23]) were tested for heterologous expression. Among them, the mammalian vasopressin receptor (V2R) was one of the best expressing test cases (>1 µmol/mg MP; Table 1, Figure 1C). V2R is involved in the regulation of water homeostasis by the kidney and in X-linked nephrogenic diabetes insipidus [24]. The expression level of V2R in PRCs is higher than the best ones previously reported using conventional overexpression systems optimized for eukaryotic MPs [4], [25]. Human CCR5, a chemokine receptor currently serving as a major therapeutic target against HIV cell-entrance [26], was expressed at levels similar to *Drosophila* Rh1 (555 pmol/mg MP; Table 1). These examples suggest that heterologous expression in the fly eye can be applied to most class A GPCRs. Since fly Rh1 is the predominant MP in rhabdomere membranes [27], it is remarkable that the overexpression of recombinant MPs did not affect the amount of endogenous Rh1 as analyzed by Western blot (not shown). On the other hand, the high level of endogenous Rh1 does not seem to limit the expression of recombinant MPs. The rhabdomere membranes appear to have seemingly unsaturable capacity to accommodate MPs.

**Table 1:** Expression levels of target MPs.

Target MP_ *Species*	Expression level [pmol/mg total MP]	
**GPCRs** (7 TMs)		
**Endogenous Rh1**_*Drosophila*	rhodopsin	272–544*
**Rh1**_*Drosophila*	rhodopsin	502
**V2R**_*Human*	vasopressin receptor	>1000
**CCR5**_*Human*	chemokine receptor	555
**DmGluRA**_*Drosophila*	metabotropic glutamate receptor	226
**mGluR5**_*Rat*	metabotropic glutamate receptor	192
**Channel** (7 TMs)		
**ChR2** **_*Clamydomonas*	channelrhodopsin	206
**Transporters** (12TMs)		
**SERT**_*Drosophila*	serotonin transporter	493
**SERT**_*Human*	serotonin transporter	220
**EAAT2**_*Human*	glutamate transporter	173
**EAAT1**_*Drosophila*	glutamate transporter	716

*Endogenous Rh1 rhodopsin levels are 3 to 6×10^7^ Rh1 molecules/rhabdomere [20], [21] corresponding to 272 to 544 pmol/mg total MP.

**MPs were expressed under the control of the GMR^1104^ driver except for ChR2 (GMR^9146^).

### A rhodopsin knock-down is not required for high expression levels

**Figure 2:**
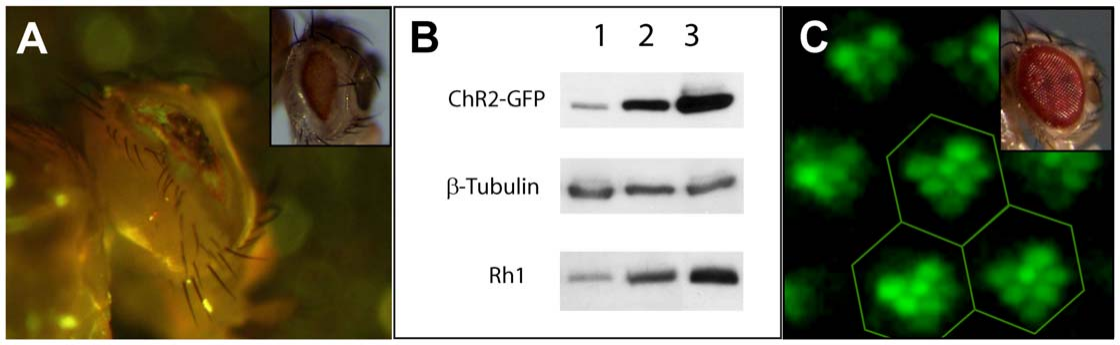
High expression levels of Channelrhodopsin ChR2 correlate with endogenous rhodopsin (Rh1). (**A**). Fluorescence image of a fly expressing ChR2-GFP under the control of the GMR^1104^ driver (Inset: bright light picture of the head). (**B**). ChR2-GFP expression driven by different GMR promoter elements (GMR^1104^ (1), GMR^9146^ (2), and GMR^8605^ (3)) analyzed by Western blot and compared with endogenous Rh1 levels. ChR2-GFP, Rh1 and β-tubulin were detected with GFP, Rh1 and β-tubulin antibodies, respectively. (**C**). Analysis of an intact head using a water-immersion objective shows rhabdomere localization of ChR2-GFP expressed under the control of GMR^9146^. Magnification was 10×20. Inset: the bright light picture shows normal eye morphology. For easier recognition, the facettes of the fly eye are marked by hexagons.

The capacity of the PRCs to host large amounts of recombinant MPs in the presence of endogenous Rh1 indicates that there is no need to down-regulate Rh1 in order to increase the expression levels. In contrary, a fly knock-out for Rh1 would alter the biogenesis of the rhabdomere membrane [28], [29]. Moreover, the expression of algal channelrhodopsin ChR2 which contains retinal as a cofactor [30] was shown to directly correlate with the levels of endogenous Rh1 (Figure 2). *Chlamydomonas reinhardtii* ChR2 was expressed under the control of different drivers including GMR drivers of diverse origins. Briefly, the use of a GMR driver (Bloomington #1104) [31] constructed on a *gl^60j^* genetic background missing the glass protein [32] and therefore Rh1 [33] gave a surprisingly strong eye-phenotype (Figure 2A) not seen i.e. for V2R-expressing flies (not shown), and ChR2 was barely detectable (Figure 2B, lane 1). Two other GMR drivers (Bloomington #9146 and #8605) expressing higher amounts of Rh1 (Figure 2B, lane 2 and 3, respectively) induced also a higher expression of ChR2 (Figure 2B, lanes 2 and 3, respectively). A correlation with Rh1 levels was not observed for other MPs targets e.g. the V2R (not shown). Therefore, expression of Rh1 and ChR2 are somehow linked. ChR2 expression reached 200 pmol/mg MP (Table 1). In the presence of Rh1, the channel localized in the rhabdomeres (Figure 2C) and the eye morphology was normal (Figure 2C, Inset). The observed retinal (Figure S1Aand Rh1 dependence for the proper processing of recombinant ChR2 indicated that the photoreceptor cells are specially adapted for the expression of retinal-binding membrane proteins.

### Heterologous and homologous expression of glutamate receptors give similar amounts

**Figure 3:**
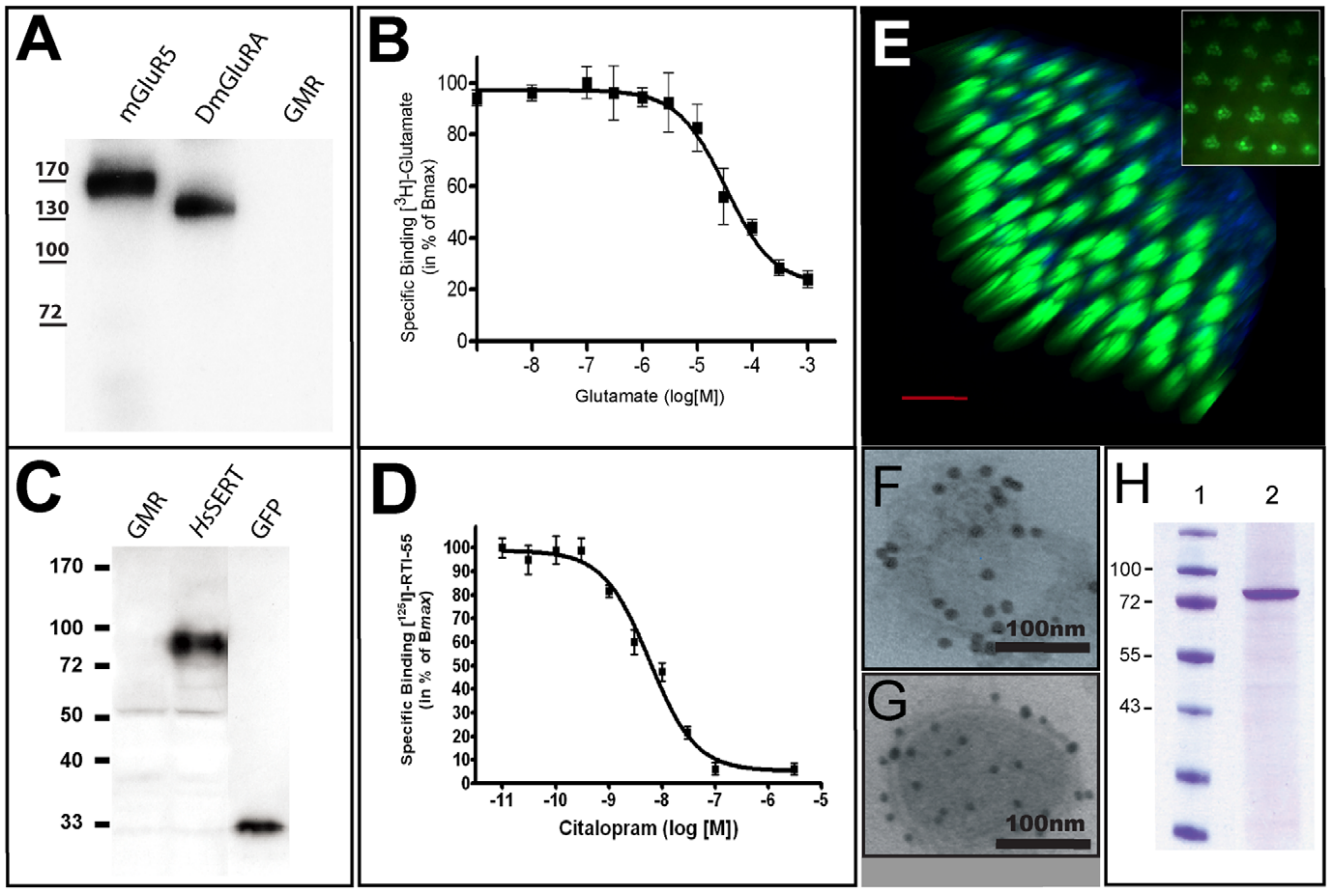
Heterologous expression of functional GPCRs and transporters in fly eyes. (**A**). Western blot analysis of *Drosophila* (DmGluRA-GFP) or mammalian (mGluR5-GFP) metabotropic glutamate receptors expressed in fly eyes using a GFP antibody. The GMR driver fly is shown as a negative control. (**B**). Homologous competitive binding experiment with glutamate [15] on membranes from flies expressing mGluR5-GFP. (**C**). Western blot analysis of the human serotonin transporter *Hs*SERT-GFP expressed in fly eyes using a GFP antibody. The GMR driver fly and GFP standard are shown as negative and positive controls, respectively. (**D**). Competition binding experiment on membranes from flies expressing *Hs*SERT-GFP. Binding of [^125^I]-RTI55 was competed with racemic citalopram (K_i_ 4.5±2.7 nM). (**E**). Three-dimensional reconstruction from laser scanning confocal microscopy images of a fly eye expressing *Hs*SERT-GFP. GFP fluorescent signal, showing the presence of *Hs*SERT in all rhabdomeres of all ommatidia (in green); natural autofluorescence delimits the surface of the eyes (in blue). The scale bar represents 40 µm. Inset: Analysis of an intact head using a water-immersion objective shows rhabdomere localization of *Hs*SERT-GFP expressed under the control of GMR^1104^. Magnification was 10×20. (**F–G**). EM-double immunogold labeling of recombinant *Hs*SERT-GFP and endogenous Rh1 with the GFP (10 nm gold) and the Rh1 (15 nm gold) antibodies, respectively, using purified rhabdomere membranes. (**F**). Typical Rh1-positive domain. (**G**). Typical *Hs*SERT-labeled domain. (**H**). Coomassie-stained SDS-PAGE of *Hs*SERT-GFP purified [52] in one step using a nickel column (lane 2). MW standards are shown in lane 1.

We have shown that GPCRs can be expressed in high amounts in the fly eyes. In order to compare heterologous and homologous expression we choose mGluRs. Mammalian mGluR5 is involved in antipsychotic medication and subject of intensive pharmacological and structural characterization [34], [35]. Expression of mGluR5 gave strong eye fluorescence (not shown) with expression levels similar to DmGluRA according to Western blot and fluorescence-scanning analyses (Figure 3A; Table 1). For functional tests fly heads were collected as previously described [16] and membranes were prepared for radioactive glutamate binding assays. mGluR5 had an affinity for glutamate (31±2 µM) (Figure 3B) in the same range as reported previously for *Dm*GluRA (54 µM) [15] suggesting proper folding of the heterologously expressed receptor. The results showed that heterologous expression of functional GPCRs was efficient and reached similar levels as homologous expression.

### Heterologous expression of neurotransmitter transporters

Encouraged by the success with heterologous expression of GPCRs and channelrhodopsin ChR2, we set out to test the fly eye system also for membrane transporters. For eukaryotic neurotransmitter transporters low level expression and heterogeneity have been reported from classical overexpression systems. The serotonin transporter (SERT) seems to require rather sophisticated overexpression systems i.e. with engineered chaperones [36]. We tested serotonin transporters from human (*Hs*SERT) and *Drosophila* (*Dm*SERT). Strong expression was detected by epifluorescence microscopy and by Western blot analysis for *Hs*SERT (Figure 3C). *Dm*SERT and *Hs*SERT expression quantified by fluorescence scanning of native gels was 493 and 220 pmol/mg MP, respectively (corresponding to 43 and 20 ng SERT/eye, respectively; Table 1). These expression levels are in range with endogenous rhodopsin. Proper folding of *Hs*SERT is indicated by binding the inhibitors R,S-citalopram (nanomolar affinity; Figure 3D) and cocaine (309±38 nM, not shown) with similar affinities as reported previously [37], [38]. Similarly, the glutamate transporters *Dm*EAAT1 and *Hs*EAAT2 both expressed well in fly eyes (Table 1). These data show that the fly eye system is suitable for heterologous and homologous expression of functional neurotransmitter transporters.

### SERT and Rh1 localize in distinct domains in the rhabdomere membrane

We have shown that despite the high quantities of endogenous Rh1, SERT is expressed in similarly high amounts. In order to test whether *Hs*SERT and Rh1 co-localize in the PRCs, *Hs*SERT localization was analyzed by 3D-laser-scanning confocal microscopy (LSCM) of the intact fly head (Figure 3E) as well as by epifluorescence microscopy using water-immersion objectives [39] (Figure 3E,inset). *Hs*SERT is expressed in all 7 rhabdomeres of each compound eye called ommatidia. The distribution of *Hs*SERT (Figure 3E) and *Dm*SERT (not shown) in the rhabdomeres is similar to rhodopsins [29], [40]. However, it was possible to separate domains containing endogenous Rh1 from those containing *Dm*SERT using an Ultra-Turrax for membrane disruption and subsequent ultracentrifugation in a density gradient (Figure S3). This suggests that Rh1 and *Dm*SERT are accommodated in separate membrane areas of the rhabdomeres. To further investigate this, electron microscopy with double gold-immunolabeling using anti-Rh1- and anti-GFP antibodies was performed on rhabdomere membranes (Figure 3F–G). 56% and 42% of the membrane structures were either positive for Rh1 (Figure 3F) or SERT-GFP (Figure 3G) respectively, and only 2% contained a Rh1/SERT-GFP mixture. Therefore, Rh1 and DmSERT indeed localize in separate membrane domains. This shows the feasibility of further analysis of the supramolecular organization of SERT and probably other recombinant proteins in rhabdomere membranes by biophysical methods like cryo-electron microscopy [41] or atomic force microscopy [42], [43].

### Large-scale purification of MP targets

Some overexpression systems like *Pichia pastoris* display often impressive levels of MP production at a small scale but expression at a larger scale is tricky and requires sophisticated devices [44]. In order to test the scalability of the fly eye system, the fly cultures were expanded (Figure S2) and *Hs*SERT was subjected to large scale purification. Fly heads were collected for membrane preparation [16]. A volume of 4 ml (2 g) frozen fly heads gave typically 45 mg of total MP with 0.5 mg *Hs*SERT (1 mg *Dm*SERT) purified routinely using an affinity column (Figure 3H). The transporters and receptors are now used for detergent optimization and crystallization trials. Taken together, the amounts obtained with the fly eye system in combination with the superior homogeneity of the protein provide the basis for further biochemical, pharmacological and structural analyses.

## Discussion

We show that the expression of eukaryotic membrane proteins in the eye of transgenic *Drosophila* is a powerful tool for the production of functional GPCRs, neurotransmitter transporters and channels. For SERT we demonstrate that the fly eye system can be scaled up to the amounts needed for routine crystallization studies and biochemical characterization. The expression levels of a number of test cases come close to that of endogenous rhodopsin. Using a GFP tag for monitoring allows for easy *in vivo* and *in vitro* MP analysis and quality control of the fly cultures.

Specific properties of the fly eye system offer major advantages compared to conventional expression systems. These include accessibility, low cost and superior quality of the expressed proteins [14], [45]. The PRCs maintain a high turnover of rhodopsin in their specialized membrane stacks [21], [46] which relies on high-throughput MP production, folding and targeting. Being specialized and polarized cells, PRCs [47], [48] harbor the rhabdomeres as an ideal storage compartment for MPs. PRC targeting of MPs that are often toxic for the host cell might benefit from the absence of endogenous ligand or from having only minor effects on local metabolism. We observed that the capacity of the PRCs to host MPs seems almost unsaturable, as in addition to endogenous rhodopsin equivalent amounts of recombinant MP can be accommodated. Heterologous expression can reach a similar level as homologous expression as shown for the mammalian mGluRs and SERT. The fly eye system is therefore particularly suited for heterologous expression.

In conventional eukaryotic expression systems ER retention of recombinant GPCRs and transporters can indicate improper folding and is often a problem e.g. for expression in yeast. In the fly eye system the majority of the target proteins were localized entirely in rhabdomere membranes. This also demonstrates that MPs with various intrinsic signal sequences are targeted to the rhabdomeres. The expression of the channelrhodopsin ChR2 was dependent on the endogenous Rh1 levels, suggesting a co-transport to the rhabdomeres. Also, there is indication that ChR2 expressed in PRCs binds its cofactor retinal, necessary for folding and activity. In addition to the classical post-translational modifications like glycosylation [49], the PRCs can efficiently produce retinal-binding proteins, while classical eukaryotic cell cultures or cell-free expression systems would require an exogenous supply of cofactor [50], [51].

Expression of MPs in the fly eye system is also a cheap alternative to expensive eukaryotic cell cultures and their requirement to work sterile. The costs for making a transgenic fly (e.g. through collaboration or using a *Drosophila* injection service) and maintaining even large scale cultures is negligible (Note: the food being made of cornmeal, moult, yeast and sugar is inexpensive with only around 10$/40 large vials). In addition, making a fly can be faster than producing baculovirus stocks for overexpression in insect cells. Due to the short life cycle of the flies, about one month is sufficient starting from the DNA-construct of the target MP to the first expression test with the transgenic fly. While an overall comparison of different expression systems is straightforward concerning the costs, the comparison of yields, workload and most importantly the protein quality requires more attention. Compared with expression systems that require liters of sterile medium, the continuous fly cultures and the handling of small volumes (125 ml of flies corresponding to 25 kflies or ≥1 mg of pure target MP) provide important advantages. When the workload of membrane preparation and the quality of the purified MPs are compared with conventional expression systems, the fly eye system is superior.

Taken together, we developed a fly eye system for the heterologous and homologous expression of different classes of eukaryotic membrane proteins. It offers a number of advantages compared to conventional expression systems and is more easily accessible than one would probably imagine. The fly eye system opens the door for studying eukaryotic membrane proteins that have so far not been accessible to biochemical and biophysical studies.

## Materials and Methods

### Cloning strategy

MP targets: the rat mGluR5 (mGluR5), human sodium-dependent serotonin transporter (*Hs*SERT), glutamate transporters (EAATs) and channelrhodopsin (ChR2) constructs were generous gifts from J.-P. Pin (Montpellier, France), R. D. Blakely (Nashville, USA), S. Birman (Marseille, France) and P. Hegemann (Berlin, Germany), respectively. The *Drosophila* melanogaster SERT (*Dm*SERT) cDNA from the Berkeley *Drosophila* Genome Project was provided by BioCat/Open Biosystems (Heidelberg, Germany).

The general protocol for cloning of target MPs has been described previously [16]. Typically, the gene coding for the target MP was amplified using EcoRI and a XhoI restriction sites and cloned in frame with eGFP (GFP) into the *Drosophila* pUAST vector [17]. GFP was flanked at the N-terminus by a Leu-Glu linker encoded by the XhoI site followed by the TEV-cleavage site ENLYFQG and at the C-terminus by a 6-his tag (TEV-GFP-6his). The construct in pUAST was sequenced and tested for expression in Schneider S2 cells as described [16].

### Transgenic fly generation

The MP-GFP construct cloned in the pUAST vector was used for classical P-element-mediated transformation of embryos [53] of the *Drosophila* host line *w^1118^* (carried out by Vanedis (Oslo, Norway) or BestGene (Chino Hills, U.S.A.)). Most of the driver lines were provided by the Bloomington center. The various driver lines used in this study were eye-specific using either the minimal rhodopsin promoter for the Rh1-GAL4 line or a glass-binding enhancer element GMR (Glass Multiple Reporter or Glass Minimum Response) for the GMR-GAL4 lines [33], [54]. The GMR driver lines used a pentameric arrangement of an enhancer region of the Rh1 promoter (glass binding site). The GMR^8506^ driver (Bloomington #8506) has a longer pentameric repeat (38 bp, “long GMR” driver) [55] than the GMR^1104^ driver (29 bp, “short GMR” driver) [31]. An advantage of the GMR^8506^ driver is that the longer enhancer site sequence confers a strict PRC specificity [31]. The ELAV-GAL4 driver (Bloomington #8765) was chosen for its predominant induction of expression in neurons [56].

Flies were reared at room temperature on standard fly food (yeast, corn syrup and agar) in a 12 hours light/12 hours dark cycle and stocks were kept at 18°C and 60% humidity. For scaling-up the fly cultures, we opted for a continuous culture in vials at room temperature instead of large cages that are difficult to handle for fly harvesting (Figure S2). For retinal depletion experiments, flies were reared for minimum two generations on carotenoid-free medium (10% yeast, 10% sucrose, 0.02% cholesterol, 2% agar) [57]. Replenishment with retinal was performed by adding 80 µg all trans-retinal on the surface of the carotenoid-free medium [49].

### Fluorescence microscopy on fly heads

For selection and sorting according to GFP fluorescence, flies were kept anaesthetized under CO2 on a glass filter (Neolab) and observed using a MZ 12-5 Leica stereomicroscope mounted with a 10× objective and equipped with an epifluorescence device (illumination path: BP 480/40 nm, dichroic mirror/reflector: 505 nm, observation path: LP 510 nm).

For rhabdomere localization experiments, flies were put asleep in CO_2_ and over-anaesthetized for 10 min in diethylether vapors, mounted on a needle and observed under water using a water-immersion objective [39] (HCX APO, L 20×/0.5 W or L 40×/0.80 W U-V-I, Leica, Germany) on a DM LFS microscope (Leica, light source: ebq 100 dc-1 [100 W], Jena GmbH, Germany; I3 filter set (illumination path: BP 450–490 nm, dichroic mirror/reflector: 510 nm, observation path: LP 515 nm)). The fluorescence was documented with a digital camera (DC200, Leica, Germany). Confocal laser scanning microscopy was performed on intact heads mounted in PBS between two coverslips spaced by clay on the stage of a Nikon TE2000-E inverted fluorescence microscope. Heads were subjected to series scan (300 z-stacks) with a 488 nm laser over half a mm depth to build a 3D-image of a whole eye.

### Harvesting of fly heads

10 ml frozen flies in liquid nitrogen were gently shaken in a 50 ml-Falcon tube together with 5 ml of glass beads (diameter 4 mm) as described [16]. Briefly, the flies and beads were transferred on a set of sieves with decreasing meshes (Neolab #6-2380 (the three smaller meshes)) pre-cooled in liquid nitrogen. After shaking, the heads were collected from the middle compartment and stored at −80°C.

### Membrane preparation

Frozen fly heads were homogenized in sucrose buffer (TRIS-HCl 50 mM, NaCl 150 mM, MgCl_2_ 2 mM, EGTA 1 mM, Sucrose 250 mM, pH 7.4) and membranes were prepared as described [16]. It is noteworthy that fly eye tissue is much easier to homogenize than cells in culture.

### Western blot analysis and quantification by fluorescence

For Western Blot, 12 µl of a sample containing 5 fly heads homogenized in 30 µl of a classical loading buffer were analyzed and detection was performed by classical enhanced chemiluminescence (ECL™, GE Healthcare) using an antibody against GFP (Biovision, Mountain View), Rh1 (4C5 ascites, DSHB, Iowa), β-tubulin (E7, DSHB, Iowa) or the *Drosophila* glutamate receptor (monoclonal 7G11 [45]).

Quantification of the fluorescent recombinant proteins was done in native gradient (4–10%)-polyacrylamide gel electrophoresis in the presence of n-Dodecyl-β-D-maltoside (DDM) or digitonin 0.1% in the gel [58]. Six fly heads were homogenized in 8 µl sucrose buffer complemented with the protease inhibitors (see membrane preparation). DDM or digitonin was added (final concentration 1%) for solubilization and left on ice for two hours. The samples were ultracentrifuged at 4°C for 10 min and 3 µl supernatant was mixed with 3 µl native loading buffer (TRIS-HCl 100 mM, glycerol 20%, Bromophenol blue). The samples were loaded in parallel with a GFP standard curve (eGFP, Biovision, Mountain View) and run at 180 V in the dark for about three hours. The gel was analyzed using the Ettan DIGE imager (GE Healthcare). Image J software was used to integrate the pixel values.

### Ligand binding

2.5 µg *Drosophila* head membranes [14] from flies expressing *Hs*SERT were incubated in 100 µl sodium phosphate buffer 50 mM, NaCl 100 mM, BSA 0.2% (pH 7.2) with [^125^I]-RTI-55 (Perkin Elmer) and increasing concentrations of racemic citalopram (Sigma) or cocaine (Sigma). Bound and free were separated by rapid filtration on a GF/B glass filter saturated with BSA 1% and polyethylene imine 0.5% using a Brandel M-48 harvester. GraphPad Prism 4.0 software was used for curve fitting and data analysis.

### Preparation of rhabdomere membranes

The eyes from 50 flies expressing *Hs*SERT were dissected and retina membranes were released using a reciprocating shaker (Mini-Bead-Beater, GlenMills, New Jersey) in the presence of 0.1 mm zirconia/silica beads (50 mg) in 125 µl ice-cold Optiprep 10%, HEPES-NaOH 10 mM, NaCl 120 mM, KCl 4 mM, sucrose 32 mM, pH 7,4 buffer. The resulting membranes were collected in the 35% Optiprep-fraction of an Optiprep-gradient (10 to 55%) after centrifugation 2.5 h at 20,000 g, 20°C. The presence of both rhodopsin and *Hs*SERT in this fraction was confirmed by Western Blot using the monoclonal 4C5 and the GFP antibody, respectively (not shown). Alternatively, the use of an ULTRA-TURRAX disperser instead of the Mini-Bead-Beater produced smaller membranes containing separated Rh1- and *Hs*SERT rhabdomere membrane sub-populations that could be recovered on a 40% and 20% Optiprep-gradient fraction, respectively (Figure S3).

### EM double immunogold labelling

Membranes resuspended in Ringer buffer at 0.1 mg/ml were adsorbed on 300-mesh carbon-coated EM grids (EM Sciences, Munich, Germany) for 2 min at RT. For immunogold labeling of GFP fusion proteins, unspecific labeling was blocked by incubating the grids on blocking solution (0.8% bovine serum albumin, 0.1% fish skin gelatin in PBS) for 10 min at RT. The samples were then double-labeled according to Slot et al. [59] except that the antibody and protein A incubation times were reduced to 15 and 10 min, respectively. The antibody against GFP (Molecular Probes, dil. 1/200) was used first followed by rhodopsin antibody (4C5, DSHB Iowa, dil. 1/1000) and rabbit anti-mouse (DaKoCytomation, Denmark). After the last incubation with protein A coupled to gold (University of Utrecht, the Netherlands), the grids were washed 5 times in PBS, 5 times in water and the samples were embedded by looping out the grids in a mixture of 8 parts methyl cellulose (Sigma, 25 centipoise; 2%) and 2 parts uranyl acetate (Fluka, Heidelberg, Germany, 3% in water) and removing excess liquid on a filter. Grids were analyzed with a Zeiss electron microscope EM10 and images taken with a Gatan MultiScan™ camera and Digital Micrograph™ software and further processed using Adobe Photoshop CS3.

### Additional methods

Additional information on large scale fly cultures is available in **Figure S2**.

## Supporting Information

Figure S1 **ChR2 expression depends on retinal.** Like rhodopsin, ChR2 is a retinal-binding protein^1^. Transgenic flies expressing ChR2-GFP grown on carotenoid-depleted food^2^, which prevents retinal synthesis, showed a clear drop in ChR2 expression (lane 2) compared to flies grown on normal medium (lane 1). ChR2 expression was recovered by replenishing the food with synthetic all-trans retinal (lane 3), indicating that the observed effect is specific for retinal. Lane 4 shows a driver fly as a control. A Western blot using a GFP antibody with two fly heads is shown. The same blot was analyzed with antibodies against β-tubulin as a control of protein load and against Rh1, respectively. The well-known dependence on retinal is observed for endogenous Rh1 expression^3^. The requirement of the chromophore for ChR2 expression could be a prerequisite for folding or could indicate that it follows the endogenous Rh1 levels.
(DOC)

Figure S2 **Large scale** ***Drosophila*** **cultures.** **1**. Initial cultures: 12 crosses (in 12 vials) were made between the *UAS-MP-GFP* fly line and the driver line in small 2,5 cm-diameter vials (10 ml fly food). Alternatively, a stable expressing line *GMR-GAL4;UAS-MP-GFP* can be used (described in^4^). **2**. Egg-laying Flies: the offspring was collected into larger vials (35 ml fly food) i.e. flies from 4 small vials transferred in one large vial with 5 cm diameter. Those flies of the first generation were used to lay eggs in large vials and were passed every fourth day in new large 5 cm-diameter vials. **3**. Harvesting Tour: the vials emptied of flies and full of larvae were used for the fly harvesting. The whole culture consisted of 12 small vials (first generation flies), around twelve larger vials used for laying eggs (first generation flies) and three racks each containing 40 large harvesting-vials (third and fourth generation flies). The time required to scale-up the culture for MP purification in milligram amounts is about one month and the culture is kept running continuously. Harvesting by flushing CO_2_ into the 3×40 vials to anaesthetize the flies and freeze them in liquid nitrogen, takes about 40 min. The harvested flies were stored at −80°C. Note: for fly harvesting vials were better than the large cages utilized for larvae collection^5^.
(DOC)

Figure S3 **Endogenous Rh1 and recombinant** ***Hs*** **SERT localize in separate rhabdomere domains.** The heads of 50 flies expressing *Hs*SERT under the control of the GMR^1104^ driver were dispersed with an Ultra Turrax in 300 µl of a buffer containing NaCl 120 mM, KCl 4 mM, sucrose 30 mM, Hepes-NaOH 10 mM pH 7.4, 8% Optiprep® and protease inhibitors (Complete®). The resulting membranes were loaded on the top of an Optiprep gradient (10 to 55%) in the same buffer, centrifuged 2.5 h at 20,000 g, 20°C and the fractions (1 to 8 from top to bottom, respectively) were analyzed by Western blot with an antibody against GFP or Rh1, respectively. The results indicate that *Hs*SERT, which localizes in rhabdomeres (Figure 3E), accumulates in different membrane areas than endogenous Rh1. *Hs*SERT-containing membranes were less dense than Rh1 domains. This difference is most likely due to the density of the membrane proteins packed in these areas.
(DOC)

Primer S1 **Primer for MP expression in fly eyes.**
(DOC)
