# Supplementary material for: Drosophila Photoreceptor Cells Exploited for the Production of Eukaryotic Membrane Proteins: Receptors, Transporters and Channels
Source: PLoS One. 2011 Apr 8;6(4):e18478. doi: 10.1371/journal.pone.0018478 (PMC3072989; doi:10.1371/journal.pone.0018478)
Supplement: Figure S1 — ChR2 expression depends on retinal. Like rhodopsin, ChR2 is a retinal-binding protein1. Transgenic flies expressing ChR2-GFP grown on carotenoid-depleted food2, which prevents retinal synthesis, showed a clear drop in ChR2 expression (lane 2) compared to flies grown on normal medium (lane 1). ChR2 expression was recovered by replenishing the food with synthetic all-trans retinal (lane 3), indicating that the observed effect is specific for retinal. Lane 4 shows a driver fly as a control. A Western blot using a GFP antibody with two fly heads is shown. The same blot was analyzed with antibodies against β-tubulin as a control of protein load and against Rh1, respectively. The well-known dependence on retinal is observed for endogenous Rh1 expression3. The requirement of the chromophore for ChR2 expression could be a prerequisite for folding or could indicate that it follows the endogenous Rh1 levels. (DOC) [file pone.0018478.s001.doc]

*Drosophila* Photoreceptor Cells Exploited for the Production of Eukaryotic Membrane Proteins: Receptors, Transporters and Channels

**Valérie Panneels, Ines Kock, Jacomine Krijnse-Locker, Meriem Rezgaoui & Irmgard Sinning**

**Supporting information: Figure S1**


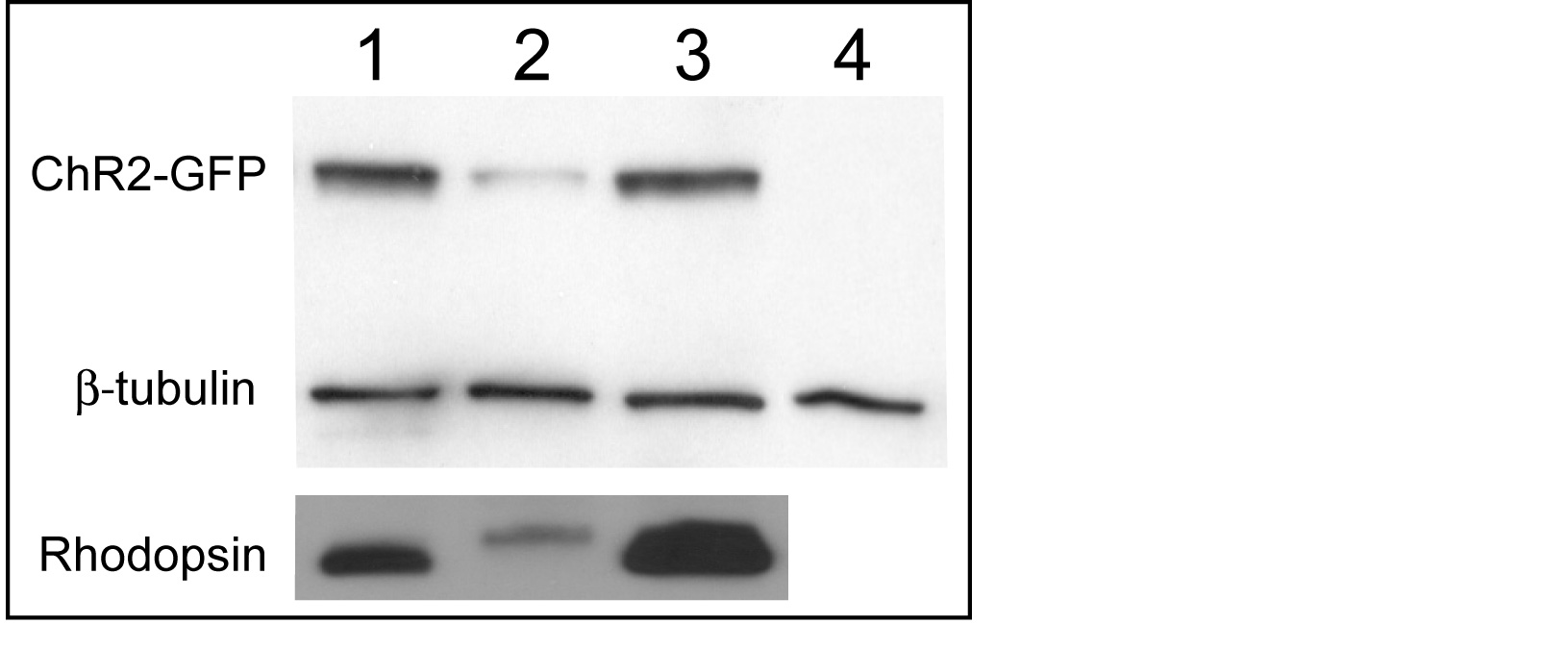


**Figure S1: ChR2 expression depends on retinal.** Like rhodopsin, ChR2 is a retinal-binding protein^1^. Transgenic flies expressing ChR2-GFP grown on carotenoid-depleted food^2^, which prevents retinal synthesis, showed a clear drop in ChR2 expression (lane 2) compared to flies grown on normal medium (lane 1). ChR2 expression was recovered by replenishing the food with synthetic all-trans retinal (lane 3), indicating that the observed effect is specific for retinal. Lane 4 shows a driver fly as a control. A Western blot using a GFP antibody with two fly heads is shown. The same blot was analyzed with antibodies against β-tubulin as a control of protein load and against Rh1, respectively. The well-known dependence on retinal is observed for endogenous Rh1 expression^3^. The requirement of the chromophore for ChR2 expression could be a prerequisite for folding or could indicate that it follows the endogenous Rh1 levels.

^1^ Nagel, G. *et al.* Channelrhodopsin-1: a light-gated proton channel in green algae. *Science.* **296** (5577), 2395-2398 (2002).

^2^ Ahmad, S.T., Natochin, M., Barren, B., Artemyev, N.O. and O'Tousa, J.E. Heterologous expression of bovine rhodopsin in Drosophila photoreceptor cells. *Invest Ophthalmol Vis Sci.* **47** (9), 3722-3728 (2006).

^3^ Harris, W.A., Ready, D.F., Lipson, E.D., Hudspeth, A.J. and Stark, W.S. Vitamin A deprivation and Drosophila photopigments. *Nature.* **266** (5603), 648-650 (1977); Ozaki, N., Rosenthal, N.E., Moul, D.E., Schwartz, P.J. and Oren, D.A. Effects of phototherapy on electrooculographic ratio in winter seasonal affective disorder. *Psychiatry Res.* **49** (2), 99-107 (1993).
