# Supplementary material for: Drosophila Photoreceptor Cells Exploited for the Production of Eukaryotic Membrane Proteins: Receptors, Transporters and Channels
Source: PLoS One. 2011 Apr 8;6(4):e18478. doi: 10.1371/journal.pone.0018478 (PMC3072989; doi:10.1371/journal.pone.0018478)
Supplement: Figure S2 — Large scale Drosophila cultures. 1. Initial cultures: 12 crosses (in 12 vials) were made between the UAS-MP-GFP fly line and the driver line in small 2,5 cm-diameter vials (10 ml fly food). Alternatively, a stable expressing line GMR-GAL4;UAS-MP-GFP can be used (described in4). 2. Egg-laying Flies: the offspring was collected into larger vials (35 ml fly food) i.e. flies from 4 small vials transferred in one large vial with 5 cm diameter. Those flies of the first generation were used to lay eggs in large vials and were passed every fourth day in new large 5 cm-diameter vials. 3. Harvesting Tour: the vials emptied of flies and full of larvae were used for the fly harvesting. The whole culture consisted of 12 small vials (first generation flies), around twelve larger vials used for laying eggs (first generation flies) and three racks each containing 40 large harvesting-vials (third and fourth generation flies). The time required to scale-up the culture for MP purification in milligram amounts is about one month and the culture is kept running continuously. Harvesting by flushing CO2 into the 3×40 vials to anaesthetize the flies and freeze them in liquid nitrogen, takes about 40 min. The harvested flies were stored at −80°C. Note: for fly harvesting vials were better than the large cages utilized for larvae collection5. (DOC) [file pone.0018478.s002.doc]

*Drosophila* Photoreceptor Cells Exploited for the Production of Eukaryotic Membrane Proteins: Receptors, Transporters and Channels

**Valérie Panneels, Ines Kock, Jacomine Krijnse-Locker, Meriem Rezgaoui & Irmgard Sinning**

**Supporting information: Figure S2**


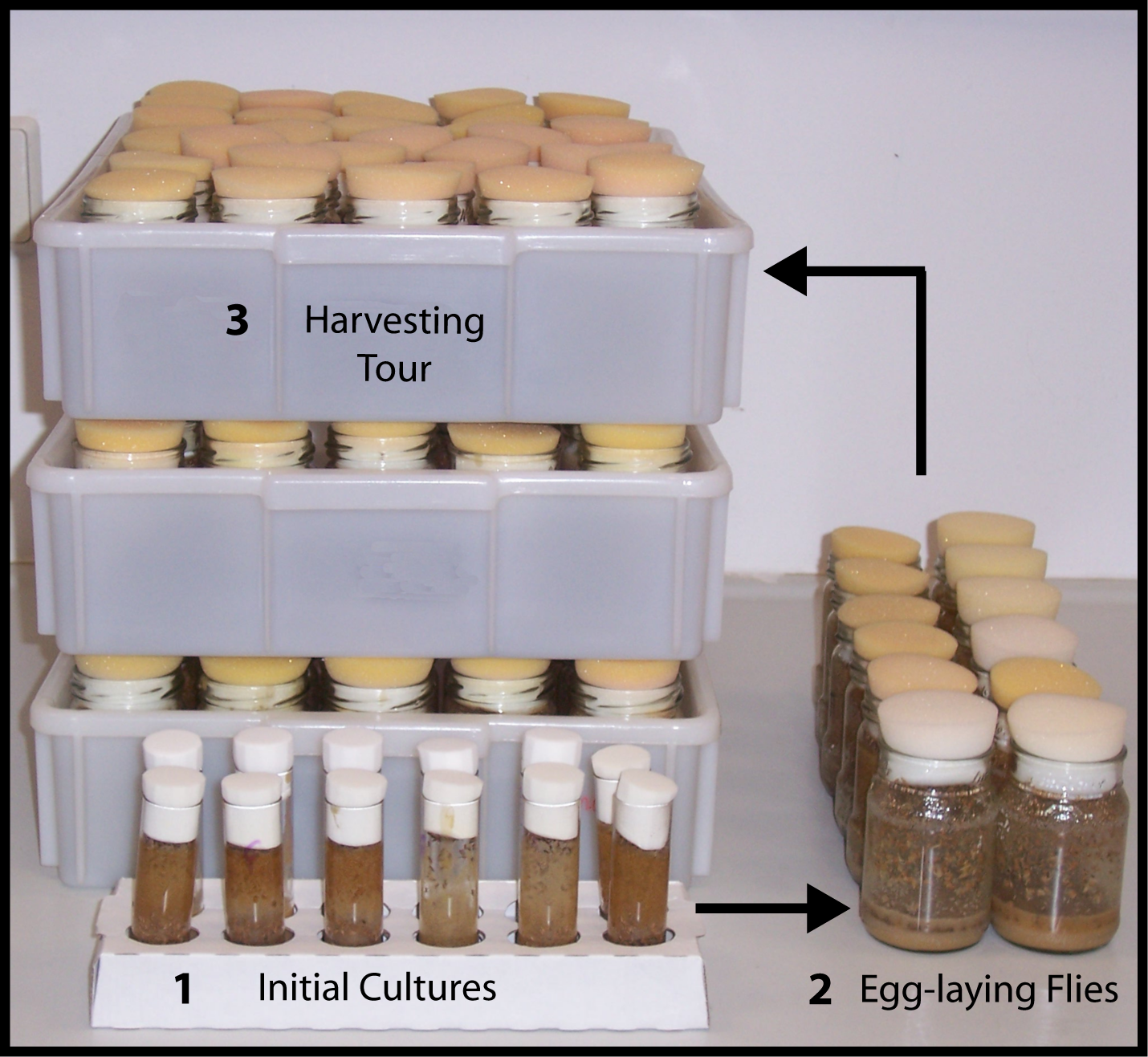


**Figure S2: Large scale *Drosophila* cultures.** **1.** Initial cultures: 12 crosses (in 12 vials) were made between the *UAS-MP-GFP* fly line and the driver line in small 2,5 cm-diameter vials (10 ml fly food). Alternatively, a stable expressing line *GMR-GAL4;UAS-MP-GFP* can be used (described in^1^). **2.** Egg-laying Flies: the offspring was collected into larger vials (35 ml fly food) i.e. flies from 4 small vials transferred in one large vial with 5 cm diameter. Those flies of the first generation were used to lay eggs in large vials and were passed every fourth day in new large 5 cm-diameter vials. **3.** Harvesting Tour: the vials emptied of flies and full of larvae were used for the fly harvesting. The whole culture consisted of 12 small vials (first generation flies), around twelve larger vials used for laying eggs (first generation flies) and three racks each containing 40 large harvesting-vials (third and fourth generation flies). The time required to scale-up the culture for MP purification in milligram amounts is about one month and the culture is kept running continuously. Harvesting by flushing CO_2_ into the 3x40 vials to anaesthetize the flies and freeze them in liquid nitrogen, takes about 40min. The harvested flies were stored at -80 ºC. Note: for fly harvesting vials were better than the large cages utilized for larvae collection^2^.

^1^ Panneels, V. and Sinning, I. Membrane protein expression in the eyes of transgenic flies. *Methods Mol Biol.* **601**, 135-147 (2010).

^2^ Kunert, N. and Brehm, A. Mass production of Drosophila embryos and chromatographic purification of native protein complexes. *Methods Mol Biol.* **420**, 359-371 (2008).
