# Supplementary material for: Drosophila Photoreceptor Cells Exploited for the Production of Eukaryotic Membrane Proteins: Receptors, Transporters and Channels
Source: PLoS One. 2011 Apr 8;6(4):e18478. doi: 10.1371/journal.pone.0018478 (PMC3072989; doi:10.1371/journal.pone.0018478)
Supplement: Figure S3 — Endogenous Rh1 and recombinant Hs SERT localize in separate rhabdomere domains. The heads of 50 flies expressing HsSERT under the control of the GMR1104 driver were dispersed with an Ultra Turrax in 300 µl of a buffer containing NaCl 120 mM, KCl 4 mM, sucrose 30 mM, Hepes-NaOH 10 mM pH 7.4, 8% Optiprep® and protease inhibitors (Complete®). The resulting membranes were loaded on the top of an Optiprep gradient (10 to 55%) in the same buffer, centrifuged 2.5 h at 20,000 g, 20°C and the fractions (1 to 8 from top to bottom, respectively) were analyzed by Western blot with an antibody against GFP or Rh1, respectively. The results indicate that HsSERT, which localizes in rhabdomeres (Figure 3E), accumulates in different membrane areas than endogenous Rh1. HsSERT-containing membranes were less dense than Rh1 domains. This difference is most likely due to the density of the membrane proteins packed in these areas. (DOC) [file pone.0018478.s003.doc]

*Drosophila* Photoreceptor Cells Exploited for the Production of Eukaryotic Membrane Proteins: Receptors, Transporters and Channels

**Valérie Panneels, Ines Kock, Jacomine Krijnse-Locker, Meriem Rezgaoui & Irmgard Sinning**

**Supporting information: Figure S3**


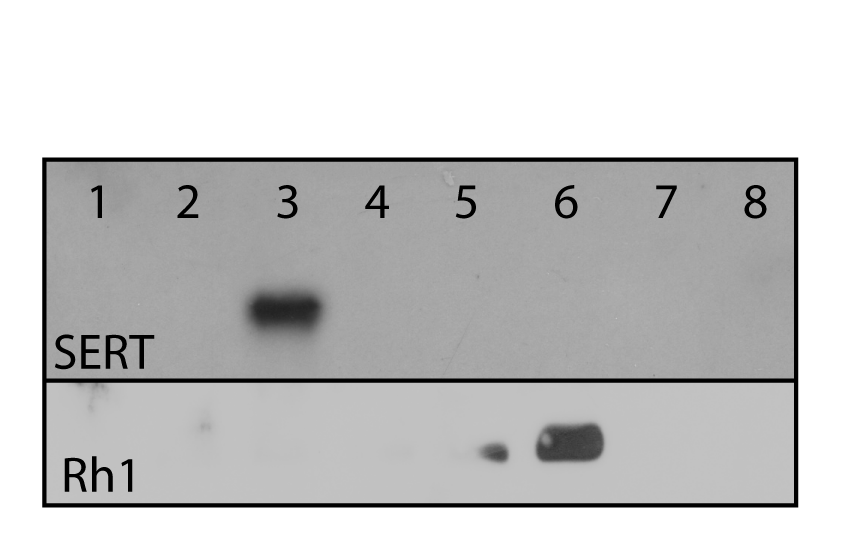


**Figure S3: Endogenous Rh1 and recombinant *Hs*SERT localize in separate rhabdomere domains.** The heads of 50 flies expressing *Hs*SERT under the control of the GMR^1104^ driver were dispersed with an Ultra Turrax in 300 μl of a buffer containing NaCl 120 mM, KCl 4 mM, sucrose 30 mM, Hepes-NaOH 10 mM pH 7.4, 8% Optiprep® and protease inhibitors (Complete®). The resulting membranes were loaded on the top of an Optiprep gradient (10 to 55%) in the same buffer, centrifuged 2.5 h at 20,000 g, 20 ºC and the fractions (1 to 8 from top to bottom, respectively) were analyzed by Western blot with an antibody against GFP or Rh1, respectively. The results indicate that *Hs*SERT, which localizes in rhabdomeres (Figure 3E), accumulates in different membrane areas than endogenous Rh1. *Hs*SERT-containing membranes were less dense than Rh1 domains. This difference is most likely due to the density of the membrane proteins packed in these areas.
