# Supplementary material for: Drosophila Photoreceptor Cells Exploited for the Production of Eukaryotic Membrane Proteins: Receptors, Transporters and Channels
Source: PLoS One. 2011 Apr 8;6(4):e18478. doi: 10.1371/journal.pone.0018478 (PMC3072989; doi:10.1371/journal.pone.0018478)
Supplement: Primer S1 — Primer for MP expression in fly eyes. (DOC) [file pone.0018478.s004.doc]

*Drosophila* Photoreceptor Cells Exploited for the Production of Eukaryotic Membrane Proteins: Receptors, Transporters and Channels

**Valérie Panneels, Ines Kock, Jacomine Krijnse-Locker, Meriem Rezgaoui & Irmgard Sinning**

**Supporting information: Primer S1**

**Primer for MP expression in fly eyes.**

Important steps in the generation of a transgenic fly expressing a target membrane protein (MP) are described below. All MPs expressed in this study are C-terminally fused to GFP for monitoring, e.g. to select of the best expressing fly, to check proper MP processing in photoreceptor cells and to control the large-scale cultures.

• The target MP is amplified by PCR with a N- or C-terminal tag. Flag-tag or his-tag at the N- or C-termini, respectively were successfully used with the fly eye system.

• The MP construct is inserted into the *Drosophila* pUAST vector either in frame with the GFP gene. A protease cleavage site can be introduced for removal of the tag.

• The protein construct is verified by sequencing and expression in S2 Schneider cells. Expression can be quickly analyzed by fluorescence microscopy or Western blot using an antibody against GFP.

• The DNA construct (30 μg MP-GFP in pUAST) is sent to a *Drosophila* injection service. “Balanced flies” are ordered to get stable transformants that went through the process of balancing with the so-called balancer chromosomes. The new fly lines (“UAS-MP-GFP”) constitute the storage culture.

• For obtaining a fly that expresses the target MP, the balanced UAS-MP-GFP flies are crossed with a driver fly from a strain driving specifically the expression to the eyes (Rh1-GAL4, GMR-GAL4, Eyeless-GAL4) using the UAS/GAL4 system.

• After 12 days at 25ºC, the flies are analyzed under a fluorescence stereomicroscope for fluorescent eyes. The eye morphology gives important evidence for choosing the best driver strain.

• For a large scale culture, 12 crossings of the UAS-MP-GFP flies are performed with the driver fly in small vials. After 12 days at 25°C the new offspring is collected in large vials. Every four days, the culture is expanded by transfering the flies into new large vials.

• Harvesting the flies: the flies, typically in a culture of 120 large vials (50 mm diameter), are anaesthetized with CO_2_ and thrown in liquid nitrogen. The fly heads are collected on sieves and stored at -80C or homogenized for membrane preparation.
